# The impact of COVID-19 on Emergency Department length of stay for urgent and life-threatening patients

**DOI:** 10.1186/s12913-022-08084-1

**Published:** 2022-05-24

**Authors:** Fengbao Guo, Yan Qin, Hailong Fu, Feng Xu

**Affiliations:** 1 Department of Emergency Medicine, the First Affiliated Hospital of Soochow University, Suzhou, Jiangsu China; 2 Clinical laboratory, the First Affiliated Hospital of Soochow University, Suzhou, Jiangsu China

**Keywords:** COVID-19, Length of Stay, resuscitation area crowding, emergency department crowding

## Abstract

**Objectives:**

To determine the impact of the Coronavirus disease-2019 (COVID-19) pandemic on the length of stay (LOS) and prognosis of patients in the resuscitation area.

**Methods:**

A retrospective analysis of case data of patients in the resuscitation area during the early stages of the COVID-19 pandemic (January 15, 2020– January 14, 2021) was performed and compared with the pre-COVID-19 period (January 15, 2019 – January 14, 2020) in the First Affiliated Hospital of Soochow University. The patients’ information, including age, sex, length of stay, and death, was collected. The Wilcoxon Rank sum test was performed to compare the LOS difference between the two periods. Fisher's Exact test and Chi-Squared test were used to analyze the prognosis of patients. The LOS and prognosis in different departments of the resuscitation area (emergency internal medicine, emergency surgery, emergency neurology, and other departments) were further analyzed.

**Results:**

Of the total 8278 patients, 4159 (50.24%) were enrolled in the COVID-19 pandemic period group, and 4119 (49.76%) were enrolled pre-COVID-19 period group. The length of stay was prolonged significantly in the COVID-19 period compared with the pre-COVID-19 period (13h VS 9.8h, *p* < 0.001). The LOS in the COVID-19 period was prolonged in both emergency internal medicine (15.3h VS 11.3h, *p* < 0.001) and emergency surgery (8.7h VS 4.9h, *p* < 0.001) but not in emergency neurology or other emergency departments. There was no significant difference in mortality between the two cohorts (4.8% VS 5.3%, *p* = 0.341).

**Conclusion:**

The COVID-19 pandemic was associated with a significant increase in the length of resuscitation area stay, which may lead to resuscitation area crowding. The influence of the COVID-19 pandemic on patients of different departments was variable. There was no significant impact on the LOS of emergency neurology. According to different departments of the resuscitation area, the COVID-19 pandemic didn’t significantly impact the prognosis of patients.

## Background

COVID-19 is acute infectious pneumonia that was declared a global pandemic by WHO in 2020 [1, 2]. COVID-19 most presents as fever and respiratory symptoms. It also affects the heart, gastrointestinal tract, liver, and other organs and can lead to multi-organ failure in severe cases. It is worth noting that many infected individuals may be asymptomatic [3, 4]. It not only seriously harms human health but also poses a challenge to international health causes. On January 15, 2020, the Chinese Center for Disease Control and Prevention (CDC) launched the first-level emergency response to the public health emergency. Medical and health institutions have actively implemented epidemic prevention and control plans, which has played an important role in controlling the spread of the epidemic. The number of patients in the emergency department is large, and the mobility is strong, which increases the difficulty for emergency medical staff. And in our hospital, the emergency department (ED) is divided into resuscitation area (accommodating the patients whose condition is urgent and life-threatening), emergency consulting room (accommodating the patients whose condition is urgent but not life-threatening), and observation room (patients who have not met hospitalization criteria but need to be observed in hospital). The resuscitation area consists of emergency internal medicine, emergency surgery, emergency neurology, and other emergency departments.

Even during the pre-COVID-19 period, the ED is often presented as the possible solution for all medical problems, leading to ED overcrowding, which is a problem that exists globally. In turn, the resuscitation area also becomes. And it isn’t easy to develop targeted solutions [5]. The resuscitation area was crowded because of the mismatch between the resuscitation area capacity and the number of patients. Therefore, the overcrowded resuscitation area may become harder to solve during the COVID-19 pandemic as the number of pressing issues increases. There is no golden standard to measure resuscitation area overcrowding [6]. The reasons for overcrowding in the resuscitation area can be summarized into three aspects: input, throughput, and output, which means the number and acuity of patients seeking treatment, patient time in the resuscitation area, and admission to the hospital or discharge [7–10]. Generally, the resuscitation area overcrowding may increase the length of stay (LOS), delay the evaluation and treatment of patients, increase the work pressure of medical staff, and increase the incidence of medical disputes and other adverse events [11].

As a "window" of the hospital, it is essential to know whether the pandemic has prolonged the LOS in the resuscitation area and caused the overcrowding of the resuscitation area. It is significant to treat patients timely and prevents the spread of the epidemic effectively in a dense crowd. Some studies have shown that emergency department visits decreased, but the LOS of resuscitation area patients increased during the COVID-19 period [12–14]. The purpose of this study was to determine the impact of the COVID-19 pandemic on the LOS and prognosis of resuscitation area patients. The objective of this study is to provide basic data that can help public health systems respond to resuscitation area crowding during a public health emergency, facilitating future clinical research and improving patient outcomes.

## Methods

### Study design

A retrospective observational study was performed. The patients’ information, including age, sex, length of stay, and death, through the resuscitation area was gathered during the COVID-19 pandemic period (January 15, 2020 – January 14, 2021) and compared with the pre-COVID-19 period (January 15, 2019 – January 14, 2020) at the First Affiliated Hospital of Soochow University. The study site is an academic hospital with nine rescue units. All ED visits are graded by trained and experienced nurses using the Taiwan Triage and Acuity Scale (TTAS). The TTAS can be divided into five grades according to the severity of illness. The lower the grade, the more critical the patient [15]. Patients with grades 1-3 need to be admitted to the resuscitation area for emergency treatment. Resuscitation area LOS was defined as the time between recorded arrival and recorded off in the resuscitation area [16].

### Statistical analysis

All measurements were tested for normality using the Shapiro-Wilk test. Continuous measurements with a normal distribution were summarized with means and standard deviations. Median and interquartile ranges (IQRs) summarize continuous measurements without normal distribution. Categorical measurements were summarized using frequencies and percentages. A Rank sum test was performed to examine the difference in the LOS between the pre-COVID-19 period and the COVID-19 period. Kruskal-Wallis tests were conducted between different months to address discrepancies between the two-time points. Fisher's Exact test was used in the emergency neurology department, and the Chi-square test was used in other departments to examine the differences in the mortality rate between the pre-COVID-19 period and the COVID-19 period. All statistical analyses were performed using R language version 4.1.0, and a *p* < 0.05 was considered statistically significant. Assumption checks were performed prior to all analyses.

### Ethics approval

This retrospective cohort study was performed in accordance with the Declaration of Helsinki and approved by the Medical Ethics Committee of the First Affiliated Hospital of Soochow University. (2021) Research Lot No. 317

## Results

### Patients characteristics

Baseline demographic clinical characteristics of patients in the resuscitation area are shown in Table 1. From January 2019 to January 2021, 8278 patients admitted to the resuscitation area were screened, with 4119 (49.76%) enrolled in the pre-COVID-19 period group and 4159 (50.24%) enrolled in the COVID-19 period group. There were no significant differences in age and gender between the two groups.

**Table 1:** Baseline demographic clinical characteristics of patients in the resuscitation area during the pre-COVID-19 period and the COVID-19 period

	pre-COVID-19 period (*n*=4119)	COVID-19 period (*n*=4159)	*p* value
Age, Median (IQR)(years)	64(48,75)	64(49,76)	> 0.05
Sex			> 0.05
Male, n (%)	2564(62.2%)	2621(63%)	
Female, n (%)	1555(37.8%)	1538(37%)	
Department			> 0.05
Emergency internal medicine, n (%)	2500(60.7%)	2516(60.5%)	
Emergency surgery, n (%)	705(17.1%)	696(16.7%)	
Emergency neurology, n (%)	842(20.4%)	883(21.2%)	
Other, n (%)	72(1.7%)	64(1.5%)	
Los, Median time (IQR)(h)	9.8 (3, 22)	13 (3.5, 23.9)	< 0.001
Mortality, n (%)	199(4.8%)	220(5.3%)	0.341

### Length of Stay

**Fig. 1:**
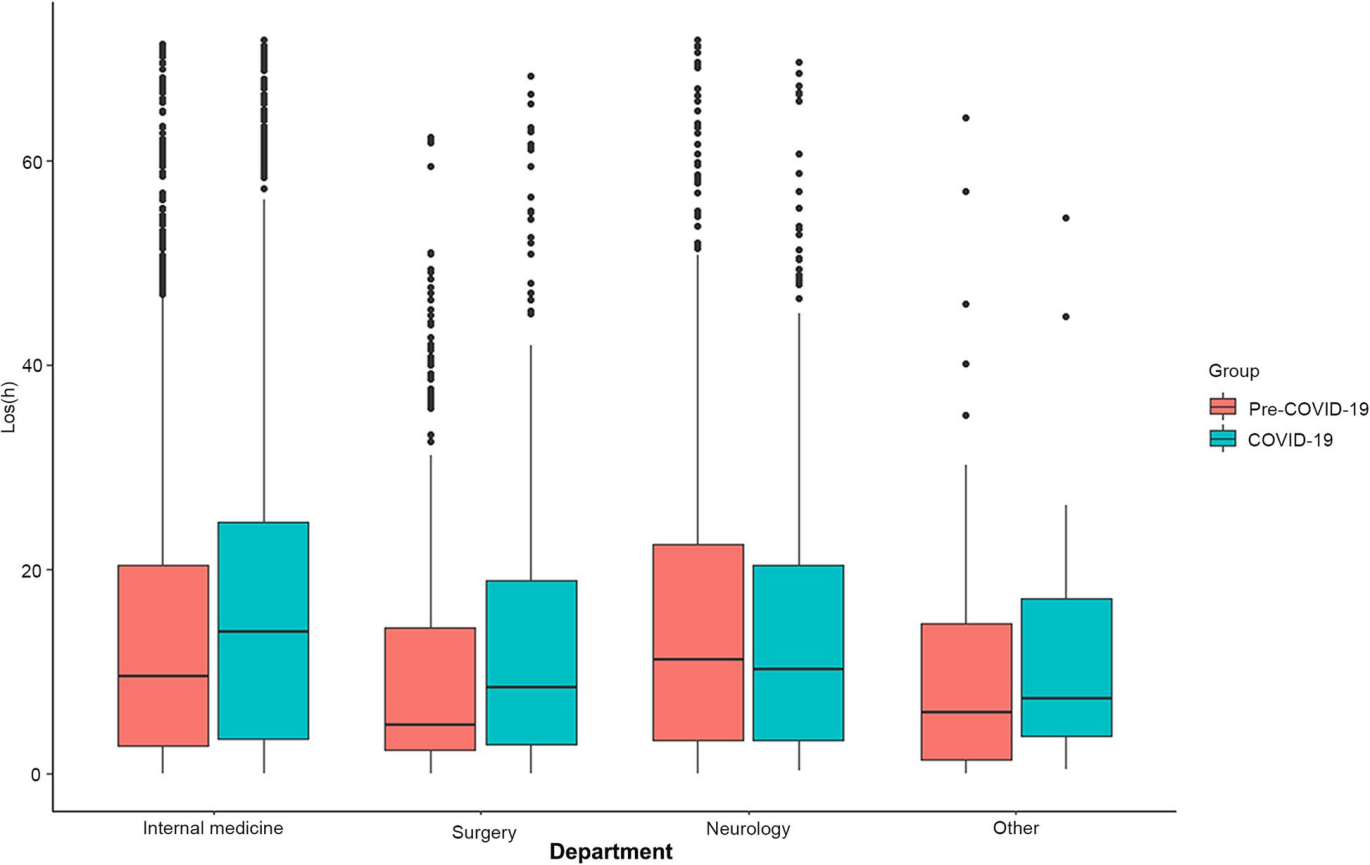
Box plot of LOS in different departments between the pre-COVID-19 period vs. the COVID-19 period

**Fig. 2:**
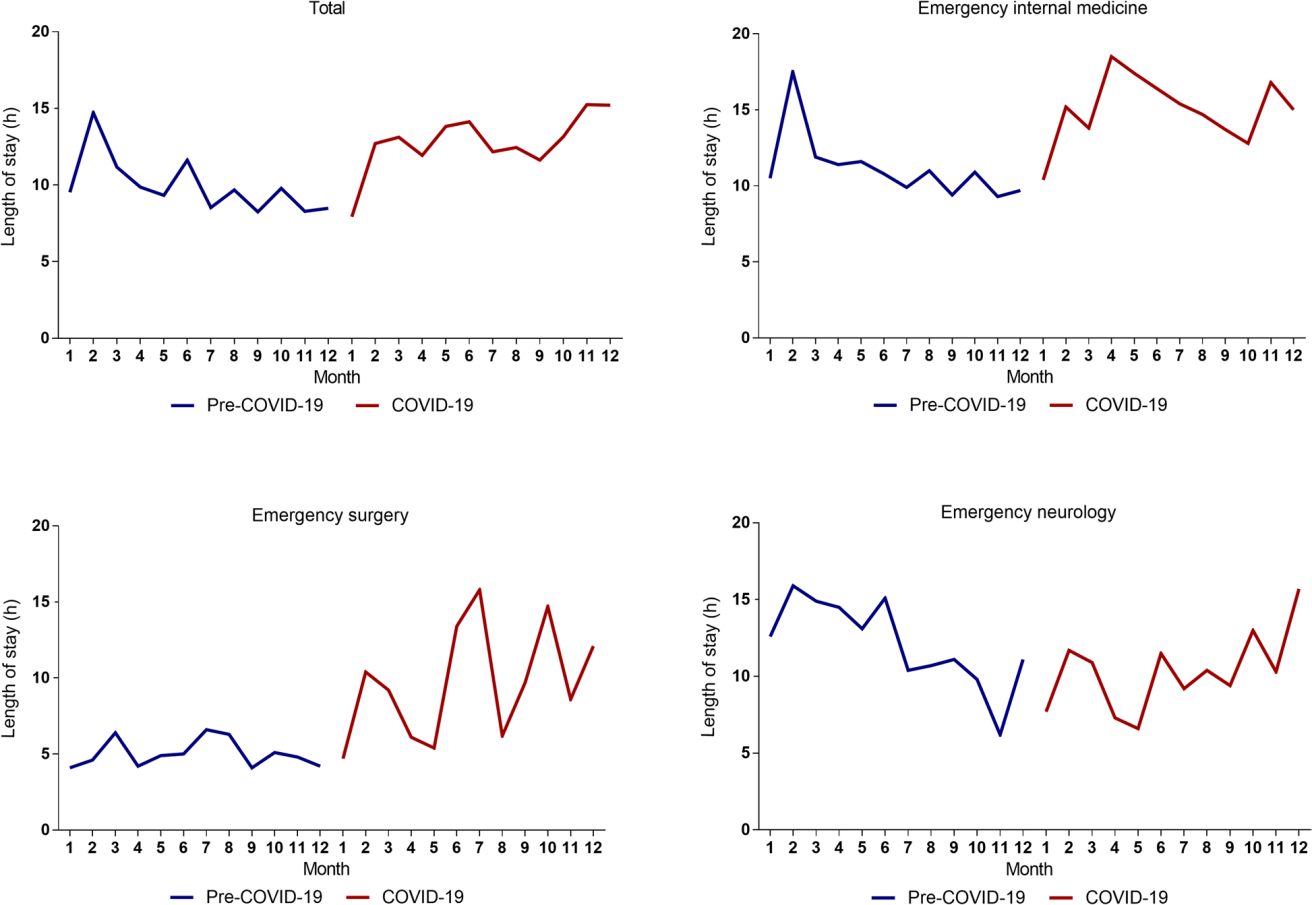
Trend of LOS between the pre-COVID-19 period and the COVID-19 period **A** The trend of LOS in all patients **B** The trend of LOS in the emergency internal medicine **C** The trend of LOS in the emergency surgery **D** The trend of LOS in the emergency neurology

There were no obvious visual differences in the number of resuscitation area visits between the pre-COVID-19 period and the COVID-19 period in emergency internal medicine, emergency surgery, emergency neurology, and other emergency departments (*p* = 0.715). The LOS was significantly longer in the COVID-19 period than in the pre-COVID-19 period (*p* < 0.001) (Table 1). The LOS median time (IQR) increased from 9.8h (3, 22) to 13h (3.5, 23.9) (*p* < 0.001). Statistical analysis of the LOS in each department (Fig. 1) showed that the LOS during the COVID-19 period was prolonged in both emergency internal medicine (15.3h VS 11.3h, *p* < 0.001) and emergency surgery (8.7h VS 4.9h, *p* < 0.001). However, the LOS of the emergency neurology department (*p* = 0.106) and else emergency department of resuscitation area (*p* = 0.084) during the pre-COVID-19 period was not statistically significant compared with the COVID-19 period. In order to show the trend intuitively, we divided the pre-COVID-19 period and the COVID-19 period into 12 months, showed the median LOS of each month, and formed a broken line graph. The LOS was statistically analyzed in each department by month, as shown in Fig. 2. The LOS increased significantly after the COVID-19 pandemic in the resuscitation area (*p* < 0.001). (Fig. 2, A) In terms of different departments of resuscitation area, the LOS in the emergency internal medicine (Fig. 2, B) and emergency surgery (Fig. 2, C) was on the rise during the COVID-19 pandemic period compared to the pre-COVID-19 period. However, there was no increasing trend in the LOS in the emergency neurology department (Fig. 2, D).

### Mortality

In this study, the number of deaths in the resuscitation area was 419 (220 during the COVID-19 period and 199 during the pre-COVID-19 period). Although the LOS during the COVID-19 period was significantly longer than the pre-COVID-19 period, there was no statistically significant relationship in the proportion of deaths between the two periods (5.3% VS 4.8%, *p* = 0.341). And there was no difference in the proportion of deaths among subgroups, including emergency internal medicine (6% VS 6.7%, *p* = 0.271), emergency surgery (3.8% VS 3.6%, *p* = 0.814), emergency neurology (2.7% VS 2.8%, *p* = 0.9), and other emergency departments (0% VS 1.6%, *p* = 0.471).

## Discussion

As an infectious disease transmission via respiratory droplet secretions or direct contact with the lesions, COVID-19 has caused a global pandemic in a short period, with a higher mortality rate than seasonal flu [17–19]. The COVID-19 genome mutations are continuously found in the course of the pandemic, and the transmission and virulence are also constantly changing with the mutations. Humanity is still in the exploratory stage of COVID-19 [20]. The COVID-19 outbreak is severely affecting the health services system worldwide [21, 22]. COVID-19 is accompanied by various symptoms; 80% of infected patients have no symptoms or mild symptoms [23]. Therefore, it is necessary for the emergency department, which is the frontline of hospitals to distinguish suspected infection patients more carefully [24]. In the current COVID-19 situation, strict screening and careful admission are necessary. Therefore, as seen in prior literature, [12–14] our study found that the length of stay in the resuscitation area was longer than before the pandemic.

There was no significant difference in the overall number of patients between the pre-COVID-19 period and the COVID-19 period in this study. Meanwhile, there was no difference in the number of patients in each department in the resuscitation area between the two periods either. However, other reports [25, 26] have shown a significant reduction in emergency department visits during the pandemic, particularly for patients with cardiovascular and neurological diseases. The main reason for reducing emergency department visits is that these patients are frightened by the risk of contagion [27]. Further research may be needed to assess the causes of the difference between our study and other reports.

**Fig. 3:**
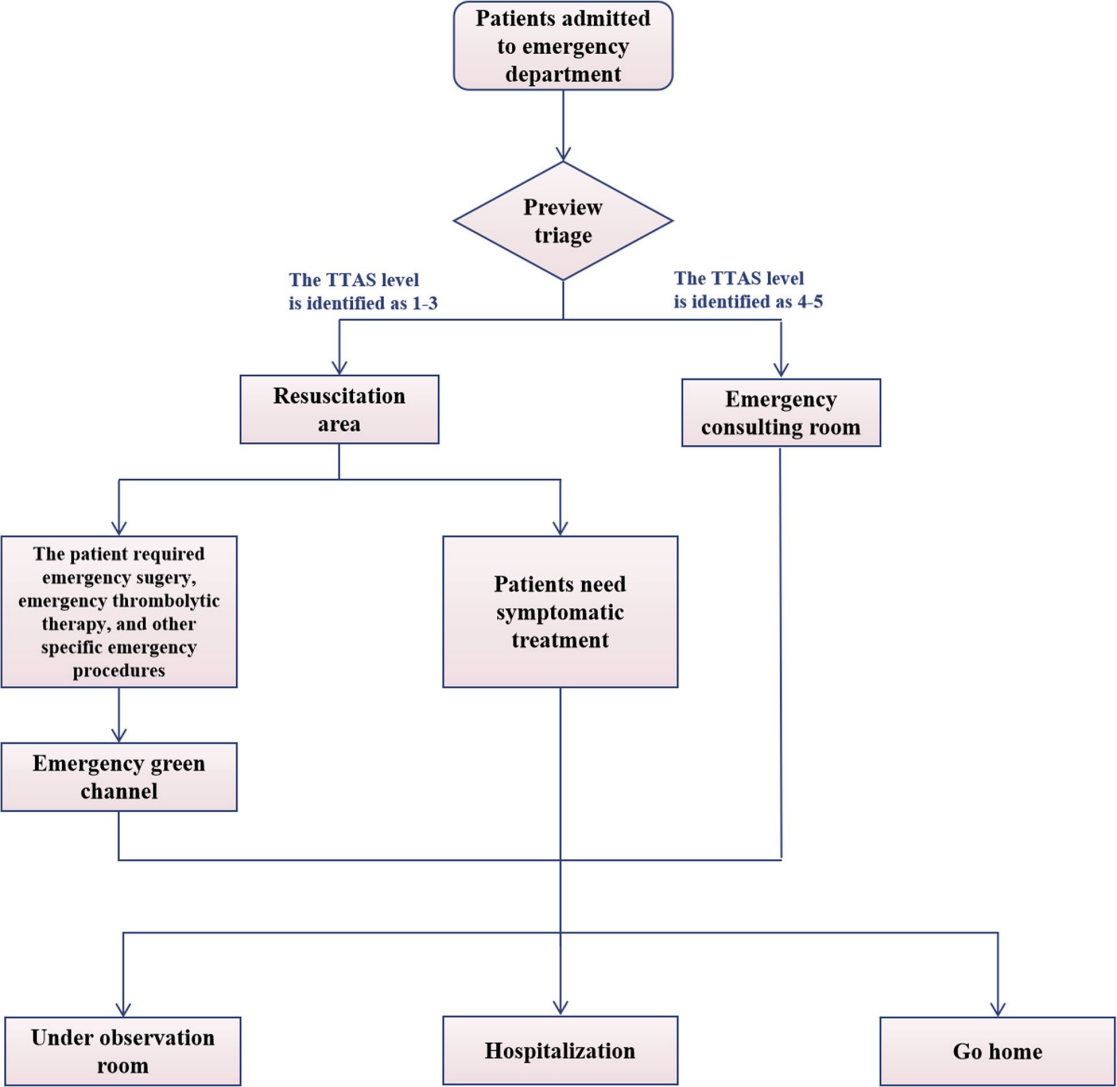
Resuscitation area workflow during pre-COVID-19

**Fig. 4:**
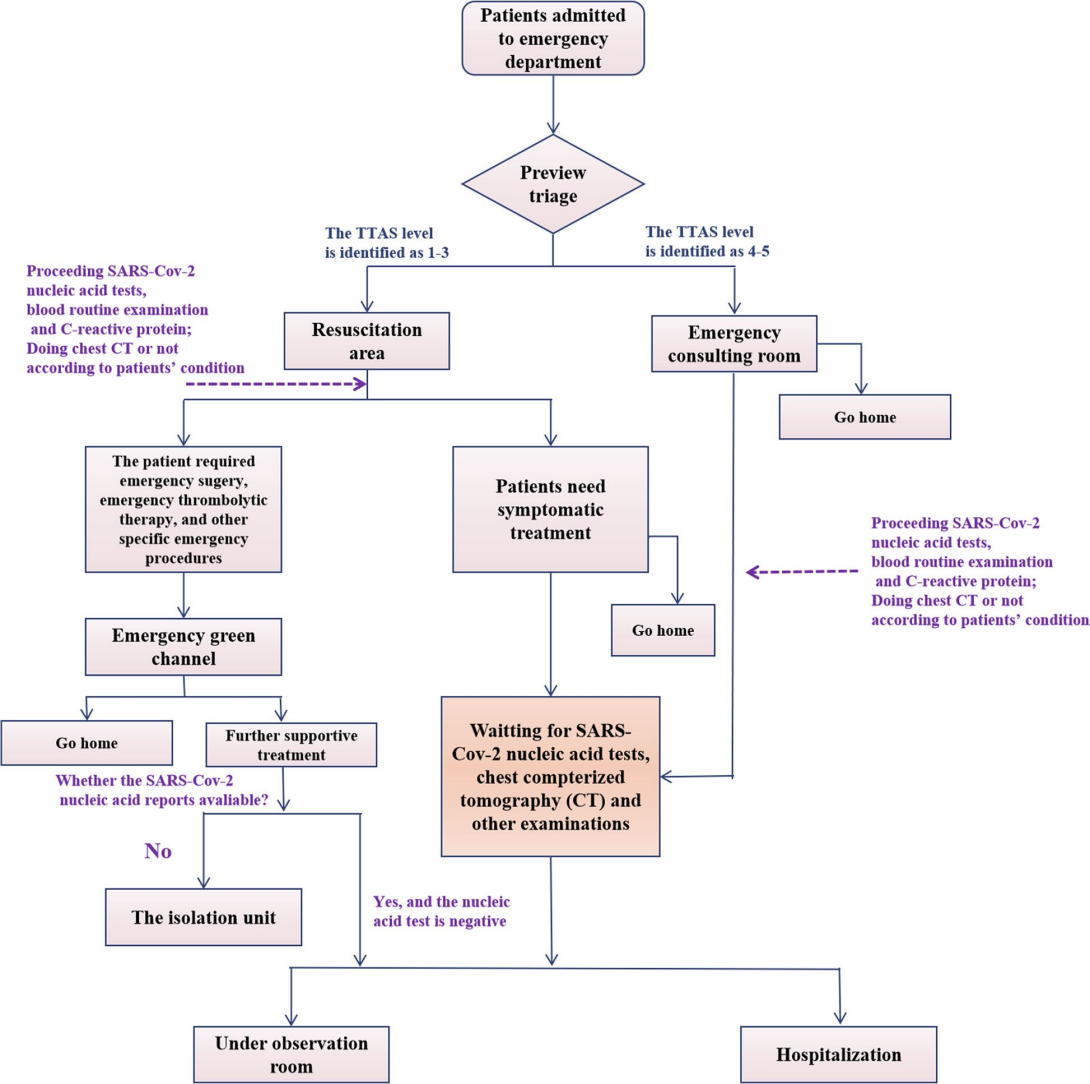
Resuscitation area workflow during COVID-19 pandemic

This study found that the LOS in the resuscitation area during the COVID-19 period was longer than in the pre-COVID-19 period. Studies in other regions have also shown the problem [12–14]. The reasons for crowding in the emergency department or resuscitation area can be divided into three aspects: input, throughput, and output. Several studies [27, 28] have shown that waiting for hospitalization is one of the main reasons for crowding and detention in the resuscitation area. Even during pre-COVID-19 periods, Fatovich et al. [29] considered that the root cause of resuscitation area overcrowding and retention lies in whether the hospital can provide enough inpatient beds. During the COVID-19 pandemic period, according to hospital regulations, patients who need to be hospitalized should provide a SARS-CoV-2 nucleic acid certificate and chest CT and other related examinations during the process of specialist admission. Compared with the pre-COVID-19 period, waiting for examination results resulted in an unavoidable delay in the time for patients to leave the resuscitation area. The resuscitation area workflows during the pre-COVID-19 period and the COVID-19 period are shown in Figs. 3 and 4. The process is more complicated during the pandemic, and the time of "throughput" is prolonged.

The important conclusion from our analysis was that the LOS was significantly prolonged in the emergency internal medicine and the emergency surgery, while the LOS was not significantly changed in the emergency neurology and other departments. Patients requiring observation or further hospitalization, whether in emergency internal medicine or emergency surgery, can only be admitted when the SARS-CoV-2 nucleic acid test results are negative, and beds are available in specialized wards, and these procedures were affected by the above reasons during the pandemic, which prolonged the LOS. However, for some acute and critical patients who need to race against time, such as trauma, intestinal obstruction, appendicitis, aortic dissection, and acute myocardial infarction, during the COVID-19 pandemic, such patients can still have access to the “emergency green channel,” which is a rapid and unblocked diagnosis and treatment strategy to save lives of the emergent and critical patients [27]. Instead of waiting for SARS-CoV-2 nucleic acid results, they can be treated in a timely way after rapid assessment by a COVID-19 assessment panel. However, patients outside the green channel must wait for SARS-COV-2 results before being hospitalized. If their reports are unavailable, they will be transferred to the isolation unit (a separate room in the ward prepared for emergency patients without negative nucleic acid test result) for further treatments. According to emergency neurology, most of the patients first diagnosed in the emergency neurology department were cerebral infarction or spontaneous intracerebral hemorrhage (ICH). Patients with severe intracerebral hemorrhage with surgical indications should be treated according to emergency surgical procedures. According to the recommended treatment strategies, [28–30] in addition to recombinant tissue plasminogen activator (rt-PA), there are few effective treatments for ischemic stroke. Still, the therapeutic window of rt-PA is 4.5 hours, beyond which the risk of cerebral hemorrhage is significantly increased. So, emergency thrombolysis for eligible patients is generally completed in the stroke unit of the resuscitation area. After the end of emergency treatment, patients will be transferred to observation or hospitalization. In this process, it is enough to wait for the SARS-CoV-2 nucleic acid results of patients. If nucleic acid results are not available after emergency treatment, patients will be transferred to isolation wards. This process of diagnosis and treatment may be the reason why the LOS of patients in the emergency neurology department was not prolonged during the COVID-19 pandemic period. But further research is needed to confirm this idea.

Several studies revealed that [31–34] the mortality increase is a common adverse consequence of resuscitation area crowding. Especially during the COVID-19 pandemic period, some patients postponed medical treatment due to fear of infection, resulting in increased morbidity and mortality [27, 35]. However, this study shows that the overall mortality rate does not change significantly during the COVID-19 pandemic period. In terms of this result, we discussed the possible causes. Generally, high mortality in the resuscitation area mainly includes acute heart disease, critically ill patients who need to be admitted to the Intensive Care Unit for monitoring and treatment, or patients who need emergency surgery. During the COVID-19 pandemic period, the hospital maintained the policy of emergency surgery and critically ill patients first and opened a green channel for critically ill patients. For example, critically ill patients with a low risk of COVID-19 infection should be admitted to subspecialized isolation wards for favorable treatment, and be released when the results are negative. Patients requiring emergency surgery were reported to the medical affairs division for surgical treatment and then isolated to a single ward waiting for inspection results and conducting further supportive therapies. Perhaps, the mortality is not increased during the COVID-19 pandemic period in the resuscitation area due to the effective implementation of these measures.

Although the study suggested that the prolonged LOS in the resuscitation area had no significant adverse effect on mortality, the crowded resuscitation area caused by the prolonged LOS still led to other negative consequences, such as patients' unsatisfactory medical treatment, patients privacy exposure, excessive work pressure of staff, medical disputes, Etc. The prolonged LOS in the resuscitation area during the COVID-19 period should be taken seriously. And the staff, resuscitation area, hospitals, and policymakers should work together to establish more effective triage mechanisms, such as increasing resuscitation area staff, improving resuscitation area rapid testing equipment, and increasing the number of isolation wards in each department in response to triage patients in resuscitation area during the COVID-19 period.

## Limitations

There are some limitations to our study. First, this study was a single-center retrospective study, and the results could not reflect the situation of other hospitals in China. Second, as the study’s retrospective nature, some information collected is limited. So, we could not further analyze the possible reasons for the prolongation of the LOS and cannot explain why the LOS seems longer from January to March. Therefore, in future studies, patients' disease severity analysis, examination time, laboratory testing capacity, and other complete information can be added to further analysis. Thirdly, since the information was missing after patients left the resuscitation area, the patients’ prognosis may be incomprehensive. Although there was no obvious change in mortality between the two periods. This result didn’t mean that the COVID-19 pandemic has no impact on the prognosis of patients with prolonged LOS in the resuscitation area. In future studies, clinical follow-up of patients leaving the resuscitation area is needed to assess their prognosis.

## Conclusion

The COVID-19 pandemic was associated with a significant increase in the length of resuscitation area stay, which may lead to resuscitation area crowding. The influence of the COVID-19 pandemic on patients of different departments was variable. There was no significant impact on the LOS of emergency neurology. According to different departments of the resuscitation area, the COVID-19 pandemic didn’t significantly impact the prognosis of patients.

## Data Availability

The data generated and analyzed in this study may not be used publicly in order to protect participants' personal information but may be obtained from corresponding authors upon reasonable request. (sz_xf@suda.edu.cn).

## Abbreviations

COVID-19: Coronavirus disease-2019
CDC: Chinese Center for Disease Control and Prevention
LOS: Length of stay
IQRs: Interquartile ranges
CT: Computerized tomography
ICH: Intracerebral hemorrhage
rt-PA: Recombinant tissue plasminogen activator
ED: Emergency department

## References

[1] The species Severe acute respiratory syndrome-related coronavirus (2020). Classifying 2019-nCoV and naming it SARS-CoV-2. Nat Microbiol.

[2] Cucinotta D, Vanelli M (2020). WHO Declares COVID-19 a Pandemic. Acta Biomed.

[3] Guan WJ, Ni ZY, Hu Y, Liang WH, Ou CQ, He JX, Liu L, Shan H, Lei CL, Hui DSC (2020). Clinical Characteristics of Coronavirus Disease 2019 in China. N Engl J Med.

[4] Atri D, Siddiqi HK, Lang JP, Nauffal V, Morrow DA, Bohula EA (2020). COVID-19 for the Cardiologist: Basic Virology, Epidemiology, Cardiac Manifestations, and Potential Therapeutic Strategies. JACC: Basic Transl Sci.

[5] Morley C, Unwin M, Peterson GM, Stankovich J, Kinsman L (2018). Emergency department crowding: A systematic review of causes, consequences and solutions. PLoS One.

[6] Moskop JC, Sklar DP, Geiderman JM, Schears RM, Bookman KJ (2009). Emergency Department Crowding, Part 1—Concept, Causes, and Moral Consequences. Ann Emerg Med.

[7] Asplin BR, Magid DJ, Rhodes KV, Solberg LI, Lurie N, Camargo CA (2003). A conceptual model of emergency department crowding. Ann Emerg Med.

[8] Ahalt V, Argon NT, Ziya S, Strickler J, Mehrotra A (2018). Comparison of emergency department crowding scores: a discrete-event simulation approach. Health Care Manag Sci.

[9] Moskop JC, Geiderman JM, Marshall KD, McGreevy J, Derse AR, Bookman K, McGrath N, Iserson KV (2019). Another Look at the Persistent Moral Problem of Emergency Department Crowding. Ann Emerg Med.

[10] Bernstein SL, Asplin BR (2006). Emergency department crowding: old problem, new solutions. Emerg Med Clin North Am.

[11] Freund Y, Goulet H, Leblanc J, Bokobza J, Ray P, Maignan M, Guinemer S, Truchot J, Féral-Pierssens AL, Yordanov Y (2018). Effect of Systematic Physician Cross-checking on Reducing Adverse Events in the Emergency Department: The CHARMED Cluster Randomized Trial. JAMA Intern Med.

[12] Singh S, Koirala B, Thami R, Thapa A, Thapa B, Kayastha A, Dahal P (2021). Length of Stay in the Emergency Department during COVID-19 Pandemic in a Tertiary Care Hospital: A Descriptive Cross-sectional Study. JNMA J Nepal Med Assoc.

[13] Lucero A, Sokol K, Hyun J, Pan L, Labha J, Donn E, Kahwaji C, Miller G (2021). Worsening of emergency department length of stay during the COVID-19 pandemic. J Am Coll Emerg Physicians Open.

[14] Xiong X, Wai AKC, Wong JYH, Tang EHM, Chu OCK, Wong CKH, Rainer TH (2021). Impact of varying wave periods of COVID-19 on in-hospital mortality and length of stay for admission through emergency department: A territory-wide observational cohort study. Influenza Other Respir Viruses.

[15] Ng CJ, Yen ZS, Tsai JC, Chen LC, Lin SJ, Sang YY, Chen JC (2011). Validation of the Taiwan triage and acuity scale: a new computerised five-level triage system. Emerg Med J.

[16] Simkhada P, Acharya S, Lama R, Dahal S, Lohola N, Thapa A (2020). Emergency Stay Duration of Patients in Emergency Department of A Tertiary Care Hospital in Nepal: A Descriptive Cross-sectional Study. JNMA J Nepal Med Assoc.

[17] Arellanos-Soto D, Padilla-Rivas G, Ramos-Jimenez J, Galan-Huerta K, Lozano-Sepulveda S, Martinez-Acuña N, Treviño-Garza C, Montes-de-Oca-Luna R, Rivas-Estilla AM (2021). Decline in influenza cases in Mexico after the implementation of public health measures for COVID-19. Sci Rep.

[18] Fowlkes A, Steffens A, Temte J, Lonardo SD, McHugh L, Martin K, Rubino H, Feist M, Davis C, Selzer C (2015). Incidence of medically attended influenza during pandemic and post-pandemic seasons through the Influenza Incidence Surveillance Project, 2009-13. Lancet Respir Med.

[19] Herbstreit F, Overbeck M, Berger MM, Skarabis A, Brenner T, Schmidt K (2021). Characteristics of Critically Ill Patients with COVID-19 Compared to Patients with Influenza-A Single Center Experience. J Clin Med.

[20] Wiersinga WJ, Rhodes A, Cheng AC, Peacock SJ, Prescott HC (2020). Pathophysiology, Transmission, Diagnosis, and Treatment of Coronavirus Disease 2019 (COVID-19): A Review. JAMA.

[21] Kim HS, Jang TC, Kim GM, Lee SH, Ko SH, Seo YW (2020). Impact of the coronavirus disease 2019 outbreak on the transportation of patients requiring emergency care. Medicine (Baltimore).

[22] Emanuel EJ, Persad G, Upshur R, Thome B, Parker M, Glickman A, Zhang C, Boyle C, Smith M, Phillips JP (2020). Fair Allocation of Scarce Medical Resources in the Time of Covid-19. N Engl J Med.

[23] Wu Z, McGoogan JM (2020). Characteristics of and Important Lessons From the Coronavirus Disease 2019 (COVID-19) Outbreak in China: Summary of a Report of 72 314 Cases From the Chinese Center for Disease Control and Prevention. JAMA.

[24] Oh YJ, Kim GM, Ko SH, Seo YW, Lee SH, Jang TC (2021). Effects of dynamic response to coronavirus disease outbreak in a regional emergency medical center: A retrospective study. Medicine (Baltimore).

[25] Bjørnsen LP, Næss-Pleym LE, Dale J, Laugsand LE. Patient visits to an emergency department in anticipation of the COVID-19 pandemic. Tidsskr Nor Laegeforen. 2020;140(8):1–5. 10.4045/tidsskr.20.0277 32463204

[26] Kambouri K, Skarentzos K, Oikonomou P, Papachristou E, Aggelidou M (2021). Reduction in Pediatric Surgery's Emergency Department Visits During COVID-19 Pandemic in a Tertiary University General Hospital in Greece. Cureus.

[27] Ojetti V, Covino M, Brigida M, Petruzziello C, Saviano A, Migneco A, Candelli M, Franceschi F (2020). Non-COVID Diseases during the Pandemic: Where Have All Other Emergencies Gone?. Medicina (Kaunas).

[28] Feske SK (2012). Thrombolytic therapy of acute stroke. Circulation.

[29] Shinozuka K, Dailey T, Tajiri N, Ishikawa H, Kim DW, Pabon M, Acosta S, Kaneko Y, Borlongan CV (2013). Stem Cells for Neurovascular Repair in Stroke. J Stem Cell Res Ther.

[30] Chen C, Wang Y, Yang GY (2013). Stem cell-mediated gene delivering for the treatment of cerebral ischemia: progress and prospectives. Curr Drug Targets.

[31] Miró O, Antonio MT, Jiménez S, De Dios A, Sánchez M, Borrás A, Millá J (1999). Decreased health care quality associated with emergency department overcrowding. Eur J Emerg Med.

[32] Richardson DB (2006). Increase in patient mortality at 10 days associated with emergency department overcrowding. Med J Aust.

[33] Sprivulis PC, Da Silva JA, Jacobs IG, Frazer AR, Jelinek GA (2006). The association between hospital overcrowding and mortality among patients admitted via Western Australian emergency departments. Med J Aust.

[34] Begley CE, Chang Y, Wood RC, Weltge A (2004). Emergency department diversion and trauma mortality: evidence from houston, Texas. J Trauma.

[35] Bodilsen J, Nielsen PB, Søgaard M, Dalager-Pedersen M, Speiser LOZ, Yndigegn T, Nielsen H, Larsen TB, Skjøth F (2021). Hospital admission and mortality rates for non-covid diseases in Denmark during covid-19 pandemic: nationwide population based cohort study. BMJ.

